# Distribution of Triamcinolone Acetonide after Intravitreal Injection into Silicone Oil-Filled Eye

**DOI:** 10.1155/2016/5485467

**Published:** 2016-07-14

**Authors:** Ma Da, Kenneth K. W. Li, Kevin C. Chan, Ed X. Wu, David S. H. Wong

**Affiliations:** ^1^Department of Ophthalmology, Li Ka Shing Faculty of Medicine, The University of Hong Kong, Pokfulam, Hong Kong; ^2^Department of Ophthalmology, United Christian Hospital, Kwun Tong, Kowloon, Hong Kong; ^3^Departments of Ophthalmology and Bioengineering, University of Pittsburgh, Pittsburgh, PA 15213, USA; ^4^Laboratory of Biomedical Imaging and Signal Processing, Li Ka Shing Faculty of Medicine, The University of Hong Kong, Pokfulam, Hong Kong; ^5^Department of Electrical and Electronic Engineering, The University of Hong Kong, Pokfulam, Hong Kong

## Abstract

There is increasing use of the vitreous cavity as a reservoir for drug delivery. We study the intraocular migration and distribution of triamcinolone acetonide (TA) after injection into silicone oil tamponade agent during and after vitrectomy surgery* ex vivo* (pig eye) and* in vitro* (glass bottle). For* ex vivo* assessment, intraocular migration of TA was imaged using real-time FLASH MRI scans and high-resolution T2W imaging and the* in vitro* model was monitored continuously with a video camera. Results of the* ex vivo* experiment showed that the TA droplet sank to the interface of silicone oil and aqueous almost immediately after injection and remained inside the silicone oil bubble for as long as 16 minutes. The* in vitro* results showed that, after the shrinkage of the droplet, TA gradually precipitated leaving only a lump of whitish crystalline residue inside the droplet for about 100 minutes. TA then quickly broke the interface and dispersed into the underlying aqueous within 15 seconds, which may result in a momentary increase of local TA concentration in the aqueous portion and potentially toxic to the retina. Our study suggests that silicone oil may not be a good candidate as a drug reservoir for drugs like TA.

## 1. Introduction

The vitreous cavity has increasingly been used as a reservoir for drugs in conjunction with pars plana vitrectomy to treat intraocular diseases [1]. For example, intravitreal administration of corticosteroids (e.g., triamcinolone acetonide, TA) becomes more and more popular to inhibit choroidal neovascularization [2] or to decrease inflammation [1, 3, 4]. However, effective postvitrectomy drug treatments depend on the intraocular distribution of the drugs. Indeed, a previous study [5] showed that vitrectomy surgery with tamponade agent had a significant effect on intraocular drug distribution.

In clinical practice, intraocular injection of TA at different concentrations was adopted locally to suppress cell proliferation [6–9]. It was shown that TA concentration could stay at a certain level for a long period (up to 3 months) after injection [6]. However, risk factors such as intraocular pressure elevation or RPE cell damage were reported after injection of TA at high dosage [10]. For eyes undergoing vitrectomy with silicone oil as tamponade agent, the portion of aqueous in the vitreous chamber was much less than normal eyes. This would cause an even higher postoperative intraocular TA concentration after injection of TA at the same dosage. As a result, it would be crucial to understand the distribution of the TA after intravitreal injection, whether it is released in a transient or slow manner. Currently, the distribution of TA after vitrectomy surgery is inconclusive among studies. In a preclinical study by Fernandes-Cunha et al. using rabbit model, the majority of the TA injected after vitrectomy was measurable in the silicone oil and aqueous humour up to 4 weeks after intravitreal injection [11]. A clinical case report by Jonas showed a large variation of the TA concentration in silicone oil among patients after injection after vitrectomy surgery [12]. In a study using an artificial vitreous space chamber [13], when TA suspension at high concentration (200 mg/mL) was injected into the silicone oil-filled chamber, the suspension sank to the bottom of the silicone oil within the first 5 minutes and started to sediment from then on with no retrievable TA from the silicone oil at the end. However, to the best of our knowledge, there is currently no study investigating the distribution of the TA immediately after injection using animal eye models.

This study aims to investigate the intraocular migration and distribution of TA after injection into silicone oil tamponade agent (Siluron® 2000) immediately following vitreoretinal surgery. We used an* ex vivo* pig eye model in combination with MRI techniques to allow visualization and quantification of the TA droplet kinetics in an in-house assembled drug distribution kit. For assessment over a longer period of time, an* in vitro* glass bottle model was used instead, and the migration and distribution of TA were monitored with a video camera. We developed a novel method to investigate the drug kinetics immediately after administration. Based on the findings of this study, we proposed potential improvement or alternatives over current postvitrectomy drug delivery procedure, to avoid the potential toxic effects towards the pathology such as the detached retina.

## 2. Materials and Methods

Among various species, the anatomy of porcine eye, including pupil, lens, macula-like structure, and retina vasculature (which is fully vascularized, while other animals like rabbit are not), is the second closest in similarity to human eyes (right after the primates) [14–16]. As a result, pig model was used for performing the vitrectomy surgery and assessing the drug delivery.

Siluron 2000 was adopted as the intraocular tamponade in vitrectomy surgery. It is a kind of conventional silicone oils comprising pure polydimethylsiloxane. All chemicals were purchased from Sigma-Aldrich (US) unless otherwise mentioned.

### 2.1. Vitrectomy Surgery Procedure

**Figure 1:**
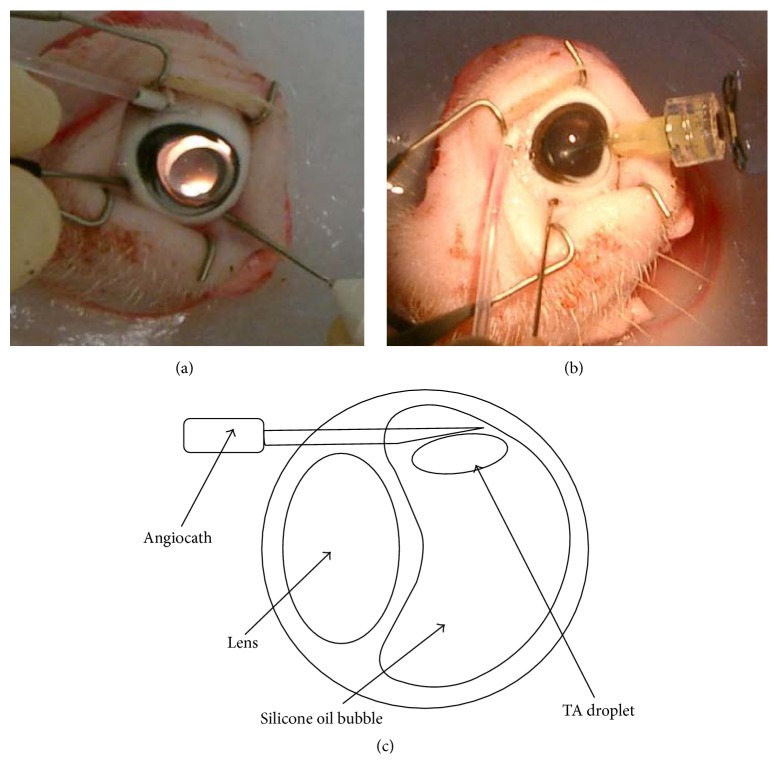
Steps of vitrectomy surgery on pig eye. (a) The vitreous was removed using a pneumatic cutting device and a light pipe (Total Plus Pak with Accurus, Alcon, US) with the help of contact lens (Ocular Instruments, US). (b) Siluron 2000 was injected after vitreous removal and air-fluid exchange. (c) Schematic diagram of the vitrectomized pig eye with an angiocath inserted into the silicone oil bubble.

Three freshly harvested pig eyes were obtained from the Sheung Shui slaughterhouse and kept in balanced salt solution (BSS) before surgery at 4°C. Within 3 hours after harvest, pig eyes underwent 3-port pars plana vitrectomy (PPV). The 3-port PPV was performed with an operating microscope (Leica Microsystems AG, Switzerland) and vitrectomy machine (Storz Instrument Company, US). Three small incisions with a diameter less than 2 mm were made 3 mm posterior to the corneoscleral limbus with a 20-gauge MVR blade for the insertion of an infusion cannula, a pneumatic cutting device, and a light pipe. The cutter and light pipe were positioned with a degree not smaller than 60° to avoid lens injury. Under constant infusion of BSS, as much vitreous as possible was removed while avoiding lens damage (Figure 1(a)). After the fluid-air exchange, the vitreous cavity was filled with Siluron 2000 (Figure 1(b)). After the operation, the sclerotomies were closed with 7-0 coated Vicryl sutures (ETHICON, Johnson & Johnson, US). The bottom two incisions were closed first. For the incision at the top, the infusion cannula was replaced with a 20-gauge angiocath, with its plastic tip cut at 9 mm such that the tip can enter the silicone oil bubble (Figure 1(c)). The back end of the angiocath was later connected to an extended cannula.

### 2.2. Drug Distribution Kit Assembly

**Figure 2:**
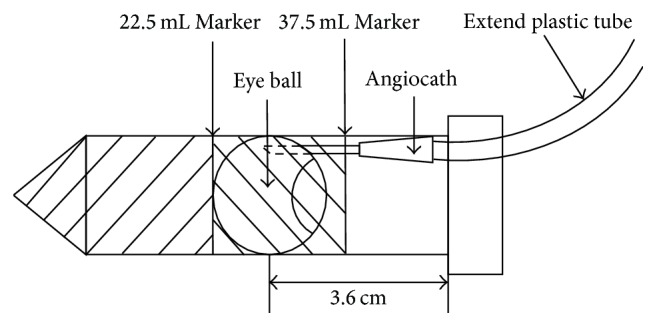
Assembly of the drug distribution kit. Step 1: 22.5 mL agarose gel was poured into the 50 mL tube. Step 2: the pig eye was placed on top of the solidified agarose gel at the upright position with the angiocath connected to the extended plastic tube. Step 3: more agarose gel was poured to cover and fix the pig eye inside the 50 mL tube.

After vitrectomy surgery, the pig eyes were placed and fixed in a self-assembled MRI compatible drug distribution kit (Figure 2), which were used to facilitate the dynamic T1-weighted MRI scan during the drug injecting. Agarose gel (2%) was poured into a 50 mL conical tube to fix the pig eye position and to avoid random movement. The position of the pig eyes was adjusted to ensure the highest magnetic resonance signal inside the MRI machine. A 1 m long plastic tube filled with the TA suspension was connected to the angiocath. Air bubbles were carefully eliminated to avoid any potential artefact during the subsequent MRI acquisitions. The eyeball was then placed in the 50 mL conical tubes containing solidified agarose and stationed in the upright position. Additional melted agarose gel was then poured into the tube until the entire eyeball was covered. A hole of 2 cm diameter was drilled at the centre of the cap in order to let the extended plastic tube come through (Figure 2(c)). The tube was then connected to a 1 mL syringe. The assembled drug distribution kit was then inserted into the phantom holder of the MRI machine.

### 2.3. Magnetic Resonance Imaging

MRI scan was performed within one hour after the vitrectomy surgery using a 7-Tesla MR system (70/16 PharmaScan, Bruker BioSpin GmbH, Germany) and a volume transmit-receive head coil. The drug injection and distribution processes were recorded in real time with T1-weighted MRI for 6 minutes. Meanwhile, high-resolution T2-weighted MRI was also taken at time points immediately before drug injection and 5 minutes after the completion of T1-weighted MRI scan, for the purpose of comparing the intraocular environment before and after drug injection.

The scanning parameters of the T1-weighted and T2-weighted MRI are summarised in Table 1.

After putting the drug distribution kit into the MRI machine, the first T2-weighted MRI scan was performed. The T1-weighted MRI scan started immediately after that. The coronal scanning orientation was selected as it can reveal the drug droplet sinking procedure clearly. The coronal axle of both the T2-weighted MRI scan and T1-weighted MRI scan was adjusted to the upright position in order to keep the measurements of the sinking speed accurate and comparable to each other. 0.1 mL TA suspension was injected into the silicone oil bubble within 10 seconds. Subsequently, the T1-weighted MRI continued to record the sinking and sedimentation processes of the drug droplet for up to 6 minutes. Five minutes after the completion of the T1-weighted MRI scan, another T2-weighted MRI scan was performed.

To quantify the intraocular kinetics of the TA droplet inside the silicone oil bubble, we measured the distance between the TA droplet and the bottom of the silicone oil bubble along time after TA injection. Each distance was calculated as the average of three measure separate measurements. The sinking speed of the drug droplet was then measured from the acquired image series by calculating its position difference from images acquired at different time points.

### 2.4. *In Vitro* Investigation of Drug Distribution

In order to further study the distribution of the TA droplet in silicone oil for an extended period of time,* in vitro* studies were performed in 20 mL glass bottles (2.5 cm in diameter) as a pseudo eye model. We hypothesized that TA suspension with higher concentration could break the surface tension between silicone oil and aqueous more easily and so would stay inside the silicone oil bubble for a shorter time. To mimic the intraocular aqueous environment during vitrectomy, the bottle was filled with 10 mL BSS first. 10 mL Siluron 2000 was then filled and formed a silicone oil layer above the BSS layer. 0.1 mL TA suspension with different concentration (2 mg/mL, 10 mg/mL, or 40 mg/mL) was injected at the top of silicone oil layer. A video camera was used to monitor the process continuously and take pictures automatically at a time interval of 15 seconds. The sedimentation time was computed starting from the time when the drug bubble reached the interface of silicone oil and BSS and ending when it broke the surface tension and dispersed into the underlying BSS layer. The experiment was repeated 7 times and averaged. One-way ANOVA followed by Bonferroni posttest was performed to evaluate the statistical significance of the differences among different groups (Prism version 5.0, GraphPad Software, Inc., San Diego, CA). Bonferroni posttest was performed only when the means of the groups were significantly different (*P* < 0.05).

## 3. Results

### 3.1. *Ex Vivo* Investigation of Postinjection Drug Distribution

**Figure 3:**
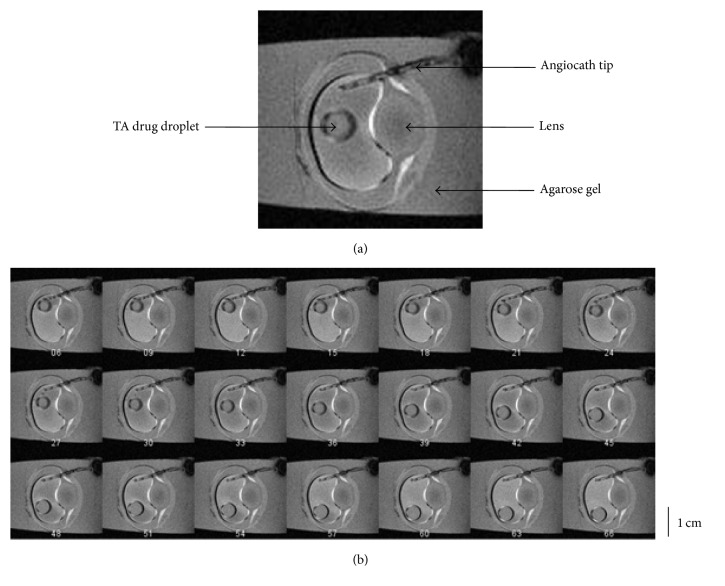
Representative image series of dynamic T1-weighted MRI. (a) The angiocath tip, the drug droplet, and the pig eye lens were clearly shown in this image frame taken with MRI. (b) MR Image series showed the intraocular kinetics of a TA droplet inside the silicone oil bubble within 6 minutes after the injection. The index number under each image represents its corresponding time point after the image acquisition starts. There are in total 72 time points within each scanning session. At each time point, the T1-weighted MRI is acquired in 5 seconds.

From the T1-weighted MRI images in the* ex vivo* study, it was observed that the TA droplet did not come out of the needle until 10 to 20 seconds after the injection started. Then it entered the silicone oil bubble within 1 second. Presumably, this was due to the viscosity of the silicone oil inside the headmost of the needles that blocked the drugs from exiting the needlepoint. Subsequently, the TA suspension showed rapid gravitation and sedimentation to the lower part of the silicone oil bubble.  Figure 3 showed the image series from the time when 0.1 mL TA droplet was fully injected into the silicone oil bubble until it sank onto the interface of the silicone oil bubble and the underlying aqueous layer. The number under each image was its frame number during real-time recording. According to the T1-weighted MRI parameter setup, the time interval between adjacent frames was 5 seconds.  Figure 3(a) showed a representative image frame which was taken at the 150th second after the commencement of T1-weighted MRI. Based on the calculation from the image frames, the average sinking speed was 0.044 mm/s. The diameter of the pig eye vitreous chamber (which was measured by the distance from top to bottom in the coronal sections images) was about 3 cm. From the MRI images, the height of the silicone oil bubble was between 1 cm and 2 cm. As a result, it could be concluded that it took about 6 minutes for the drug droplet to sink onto the interface of silicone oil and intraocular aqueous before sedimentation.

**Figure 4:**
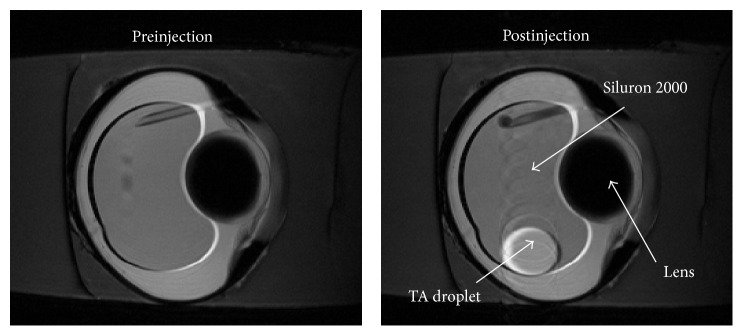
Representative T2-weighted images of vitrectomized pig eyes before and after drug injection. High-resolution T2-weighted images were taken immediately before drug injection (before injection) and 5 minutes after the completion of dynamic T1-weighted MRI scan (after injection). The scanning parameter was adjusted in order to reduce the chemical shift artifact (CSA) while maintaining the signal to noise ratio (SNR) level. The TA drug droplet was well represented in the postinjection images, which indicated that the drug droplets could sink and stay inside Siluron 2000 silicone oil bubble for up to 16 minutes after the injection. The trail of TA suspension in the postinjection image indicated the small position change of the droplet during the 5 minutes scanning time for the T2-weighted image.

The preinjection and postinjection high-resolution T2-weighted images were taken immediately before drug injection and 5 minutes after the completion of T1-weighted MRI scan (Figure 4). MRI images showed clearly that the injected TA drug droplet remained intact as a spherical droplet inside the silicone oil bubble at 16 minutes after injection (Figure 4), suggesting that TA droplets could stay inside the silicone oil bubble till the end of the experimental period in the* ex vivo* setup.

**Figure 5:**
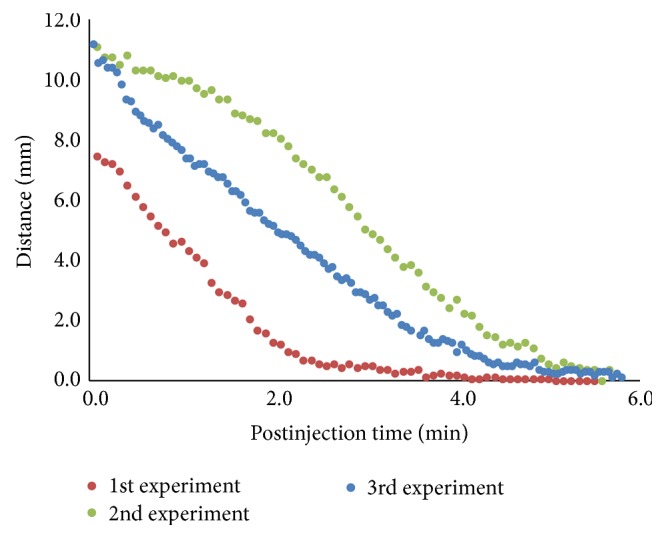
Distance from the TA droplet to the bottom of silicone oil bubble after injection. The dots in the graph represent the distance between the bottom of the TA droplet and the bottom of the silicone oil bubble along the time after injections. The 3 different colours represent 3 independent experiments performed on 3 different pig eyes. 0.1 mL TA suspension was injected into the pig eyes in each experiment. Similar to the real case scenario during postvitrectomy drug injection, the insertion sites of the angiocath tip vary among 3 experiments, resulting in slightly different kinetics of the TA droplet. The insertion site for the 1st experiment is lower than the other two, resulting in a shorter sinking time. The insertion site of the 3rd experiment is closer to the lens, resulting in a slow initial dropping speed as the TA droplet slides down along the lens before sinking straight.

The postinjection TA kinetics was measured in three independent experiments (Figure 5). The initial distance depends on the insertion position of the angiocath tip. The TA droplet sank in a linear fashion at a relatively constant speed before touching the silicone oil aqueous interface (3.23 mm/min, 3.03 mm/min, and 3.44 mm/min for each injection). The TA droplet sank to the bottom of the silicone oil bubble in 5 minutes after injection in all experiments. During the postvitrectomy drug delivery, the exact insertion site for the TA injection may vary from case to case. This is reflected in our result. The insertion site of the angiocath tip varies among the 3 experiments. For the 1st experiment, the injection site is lower than the other two, and the TA droplet sinks to the bottom of the silicone oil bubble earlier (about 3 minutes after injection, as shown in red dots in Figure 5). For the 3rd experiment, the TA droplet sank at a lower speed initially compared to the other 2 experiments (as shown in green dots in Figure 5). This is due to the fact that the initial injection site of the TA droplet is relatively closer to the lens. The TA droplet has to slide down along the lens before sinking in full speed.

### 3.2. *In Vitro* Investigation of Drug Distribution

**Figure 6:**
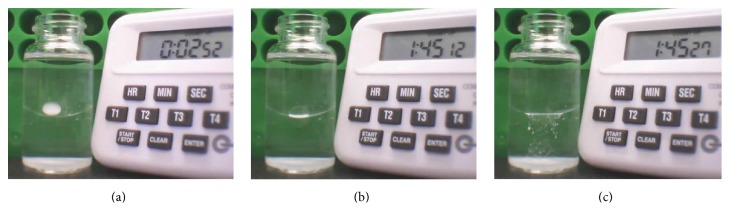
Continuous monitoring of the TA drug droplet in the 20 mL glass bottle model. This timer recorded the time elapsed after the TA droplet injection. (a) The droplet finished sinking inside the silicone oil and began to sediment. (b) The TA sediment gradually precipitated to the bottom of the drug droplet before finally breaking the surface tension of silicone oil and BSS. (c) The TA dispersed into the BSS layer after breaking the surface tension.

**Figure 7:**
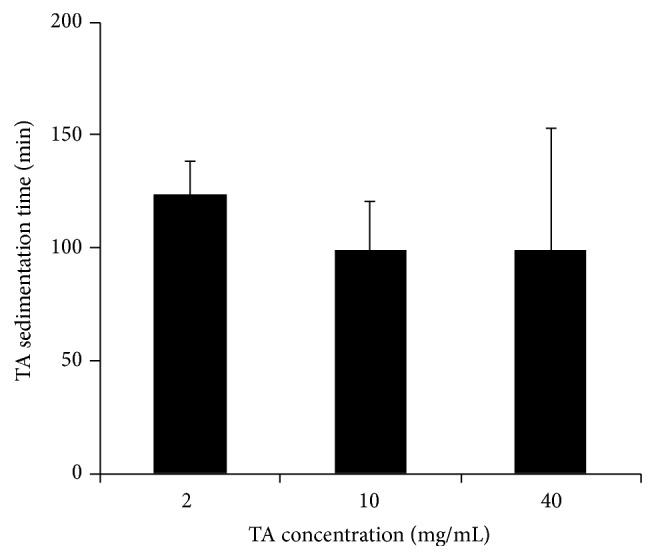
Sedimentation time of 0.1 mL TA droplet at different concentration. One-way ANOVA followed by Bonferroni posttest was performed to evaluate the statistical significance of the differences among different groups. Error bars represent standard deviation. No significant difference was found among these three groups. The variation of data from the TA droplet with a concentration of 40 mg/mL indicated the unpredictability of its sedimentation time.

The position and status of the TA droplet were further investigated using a 20 mL glass bottle in the* in vitro* study. Images were recorded every 15 seconds after TA injection into the silicone oil layer. The result showed that the TA droplet generally remained intact inside the silicone oil layer. TA gradually precipitated leaving only a lump of whitish crystalline sediment inside the droplet until about 100 minutes later when it quickly broke the silicone oil aqueous interface and dispersed into the underlying aqueous within a very short time (less than 15 seconds of the recording interval) (Figure 6). TA droplets of different concentration were used in this study and they displayed similar behaviour. The sedimentation time of TA droplet at 2, 10, and 40 mg/mL was computed and there was no significant difference among them (Figure 7). This suggested that the sedimentation time was not related to the concentration of TA in the droplet. However, the standard deviation of the sedimentation time for TA suspension at 40 mg/mL was very large, suggesting that the TA droplet at high concentration may have a more unpredictable sedimentation time.

## 4. Discussion

Ophthalmic drug release and distribution have received more and more attention in recent years [17]. Different materials and methods have been studied for drug sustainability and slow release systems [18]. For instance, some studies used collagen gel [19], while others used implant of drug surrogate [20]. Meanwhile, different vitreous substitutes have been developed and studied [21, 22], and silicone oil is the most widely used vitreous substitute currently. Clinically, TA suspension is injected after vitrectomy surgery. Therefore, it is the aim of this study to investigate the feasibility of silicone oil as a drug reservoir by evaluating the sustainability of drugs being injected.

MRI allows noninvasive and nondestructive assessments of the eye over time without depth limitation [23, 24]. The dynamic T1-weighted MRI technique is a useful tool to assist monitoring the migration and distribution of drug droplet after intraocular injection in the* ex vivo* pig eye model. Here, we showed clearly the kinetics of the drug droplet inside the silicone oil bubble, which sank to the inferior part of the silicone oil bubble. In addition, the T2-weighted MRI acquired at a later time point showed that the TA droplet stayed intact without breaking and did not mix with either silicone oil or intraocular liquid for up to 16 minutes, suggesting that the drug stayed inside the droplet without being dispersed into the silicone oil bubble. This was different from a previous study [13] where the injected TA droplet sank through the silicone oil bubble and lost its spherical shape at only 5 minutes after injection in an artificial chamber and finally sedimented below the silicone oil bubble. This difference was probably caused by the different concentration of TA between their study (200 mg/mL) and this study (40 mg/mL). Indeed, TA droplet at lower concentrations (no higher than 10 mg/mL) remained stable inside the silicone oil bubble for several hours in our* in vitro* study. However, the TA droplet status became unpredictable inside silicon oil at higher concentration. Moreover, although the T1-weighted MRI images together with the T2-weighted MRI showed an intact TA droplet inside the silicone oil bubble for at least 16 minutes, it was difficult to distinguish whether TA inside the drug droplet remained as a suspension or it precipitated out to become crystalline sediment. But both our* in vitro *study (Figure 6) and Spitzer et al.'s study [13] showed that about 90 minutes after injection only a lump of whitish TA crystalline sediment remained in the droplet. Therefore, the sustainability of TA inside the silicone oil was in question. In addition, TA sediments finally broke the silicone oil aqueous surface tension and dispersed into the underlying aqueous within a very short time, which would result in a momentary increase of local TA concentration in the aqueous portion. As it was reported that high concentration of TA had toxic effect to the retina [10], this observation suggested that silicone oil may not be a good candidate as a drug reservoir for TA.

It was interesting to observe that the TA droplet did not break the interface between silicone oil bubble and the liquid around it until almost 100 minutes later. One factor that stopped the TA droplet from breaking might be its low specific gravity. It has been shown that TA precipitation started at 5 minutes after injection [13]. Also, after comparing the sedimentation time of TA at different concentrations, we hypothesized that the reason which caused the TA droplet to finally break the oil-aqueous surface tension was the larger specific gravity and irregular shape of the crystallized TA precipitate. Whether this specific gravity difference can contribute to the silicone oil aqueous interface break needed to be further investigated. In addition, the irregular shape of TA crystals might also contribute to the surface tension break, especially for the case of unpredictable sedimentation time for TA droplet at high concentration (40 mg/mL).

The interfacial phenomena, especially the interfacial tension between vitreous substitution and aqueous, are important factors for intravitreal drug delivery. The molecules at the liquid surface possess more free energy than the molecules inside its bulk material. Therefore, both the silicone oil bubble and the TA droplet tend to reduce its surface at the interface to minimise the total energies within, resulting in a smooth interface [25]. Extra energy is needed in order to break the interface between the silicone oil and the aqueous. The energy required to increase each unit area surface is defined as the surface tension, or interfacial tension. The surface tension at the silicone oil aqueous interface is around 30 mN/m (milliNewton per meter) at room temperature [21]. The presence of solute also affects the surface tension. The solubility of TA in distilled water is very low (21 mg/L at room temperature) [26], and TA is insoluble in silicone oil [27]. This insolubility characteristic leads to the crystallized TA to sediment onto the interface between the two liquids, disrupting its smoothness and effectively increasing the surface area. Such increased surface area can also be considered as a result of the excessive energy, which comes from the gravity applied onto the crystallized TA. The tendency to release such extra energy may contribute to the final breakdown of the silicone oil aqueous interface. Methods such as dynamic surface tension measurement can be used to further analyse the liquid-liquid interfacial tension in detail [25].

Previous studies [13] showed that TA could not be retrieved within the silicone oil after intravitreal injection using an artificial vitreous chamber. It was therefore suggested that premixing the TA suspension with the silicone oil before vitrectomy would be beneficial. It would be interesting to test this method using our pig eye model. However, the TA/silicone oil suspension should be thoroughly mixed as TA and silicone oil would segregate slowly into two phases. In addition, the amount of TA to be mixed with silicone oil should be calculated in caution to avoid toxicity effect due to drug overdose. Dynamic T1-weighted MRI could be used to determine if the segregation of TA from silicone oil would still occur.

Various ocular implants have been proposed in the field of ocular drug delivery as the alternative possible vitreous substitutions to provide localised controlled drug release [28–30]. Those implants could potentially be considered as alternative drug reservoirs for TA administration after vitrectomy surgery. Some of such implants are nonbiodegradable and provide very slow and long drug kinetics, such as polyvinyl alcohol (PVA), ethylene vinyl acetate (EVA), and polysulfone capillary fibre (PF) [28]. Recently, biodegradable implants have attracted a lot of attention for sustained drug release, with the advantage of eliminating the need for surgical removal procedure. Examples of such implant include polylactic acid (PLA), polyglycolic acid (PGA), and polylactic-co-glycolic acid (PLGA) [31].

Indeed, the drug distribution characteristic in the normal eye would be worth studying in order to compare with the drug distribution in vitrectomized eyes. The* ex vivo* pig eye model and* in vitro* glass bottle model had uncontrolled IOP comparing to normal eyes. There is little research on the effect of different IOPs towards intraocular drug distribution. As a result, further investigation could be conducted to study whether IOP is a factor to influence the intraocular drug distribution inside silicone oil-filled eyes.

## 5. Conclusion

In this paper, we present a novel method to investigate the drug kinetics immediately after administration into vitrectomized eyes. We presented a self-assembled MRI compatible drug distribution kit using* ex vivo* pig eye model to monitor the sinkage of TA droplet within the bubble of silicone oil tamponade agent in real time using MRI. Furthermore, with the use of an* in vitro* pseudo eye model in a 20 mL glass bottle, we managed to monitor the postinjection drug kinetics for longer period of time and observed that the TA gradually precipitated inside the droplet and finally broke the silicone oil aqueous interface within short period of time and dispersed into the underlying fluid. The result of this study suggests that silicone oil may not be a good candidate as a drug reservoir for drugs like TA. The unpredictable sudden breakdown of liquid-liquid interface might induce transient concentration increase causing a potential postinjection toxic side effect. As a result, during the treatment of intraocular diseases such as retinal detachment with pars plana vitrectomy surgery, caution should be taken when injecting drugs (e.g., corticosteroids) into the silicone oil tamponade agent. The better alternative materials that can be both the tamponade agent and the reservoir for sustained slow drug release still remain to be investigated.

## Figures and Tables

**Table 1:** The scanning parameters of both the T1-weighted MRI and the T2-weighted MRI.

	T1-weighted MRI	T2-weighted MRI
Imaging sequence	FLASH	RARE
Repetition time (TR)	52 ms	4200 ms
Echo time (TE)	3 ms	65 ms
Slice thickness	1 mm	1 mm
Slice gap	0.1 mm	0.1 mm
Acquisition matrix	128 × 128	256 × 256
In-plane resolution	258 *μ*m × 258 *μ*m	129 *μ*m × 129 *µ*m
Field of view	3.3 mm × 3.3 mm	3.3 mm × 3.3 mm
Number of slices	5	20
RARE factor	N/A	8
Average number	1	2
Total scanning duration	6 min (5 s per time point for 72 time points)	5 min each before and after T1-weighted MRI
